# Anion–π catalysis on carbon allotropes

**DOI:** 10.3762/bjoc.19.140

**Published:** 2023-12-12

**Authors:** M Ángeles Gutiérrez López, Mei-Ling Tan, Giacomo Renno, Augustina Jozeliūnaitė, J Jonathan Nué-Martinez, Javier Lopez-Andarias, Naomi Sakai, Stefan Matile

**Affiliations:** 1 Department of Organic Chemistry, University of Geneva, Geneva, Switzerland

**Keywords:** anion–π interactions, autocatalysis, catalysis, carbon nanotubes, Diels–Alder reactions, electric-field-induced catalysis, electromicrofluidics, enolate addition, ether cyclizations, fullerenes

## Abstract

Anion–π catalysis, introduced in 2013, stands for the stabilization of anionic transition states on π-acidic aromatic surfaces. Anion–π catalysis on carbon allotropes is particularly attractive because high polarizability promises access to really strong anion–π interactions. With these expectations, anion–π catalysis on fullerenes has been introduced in 2017, followed by carbon nanotubes in 2019. Consistent with expectations from theory, anion–π catalysis on carbon allotropes generally increases with polarizability. Realized examples reach from enolate addition chemistry to asymmetric Diels–Alder reactions and autocatalytic ether cyclizations. Currently, anion–π catalysis on carbon allotropes gains momentum because the combination with electric-field-assisted catalysis promises transformative impact on organic synthesis.

## Introduction

**Figure 1:**
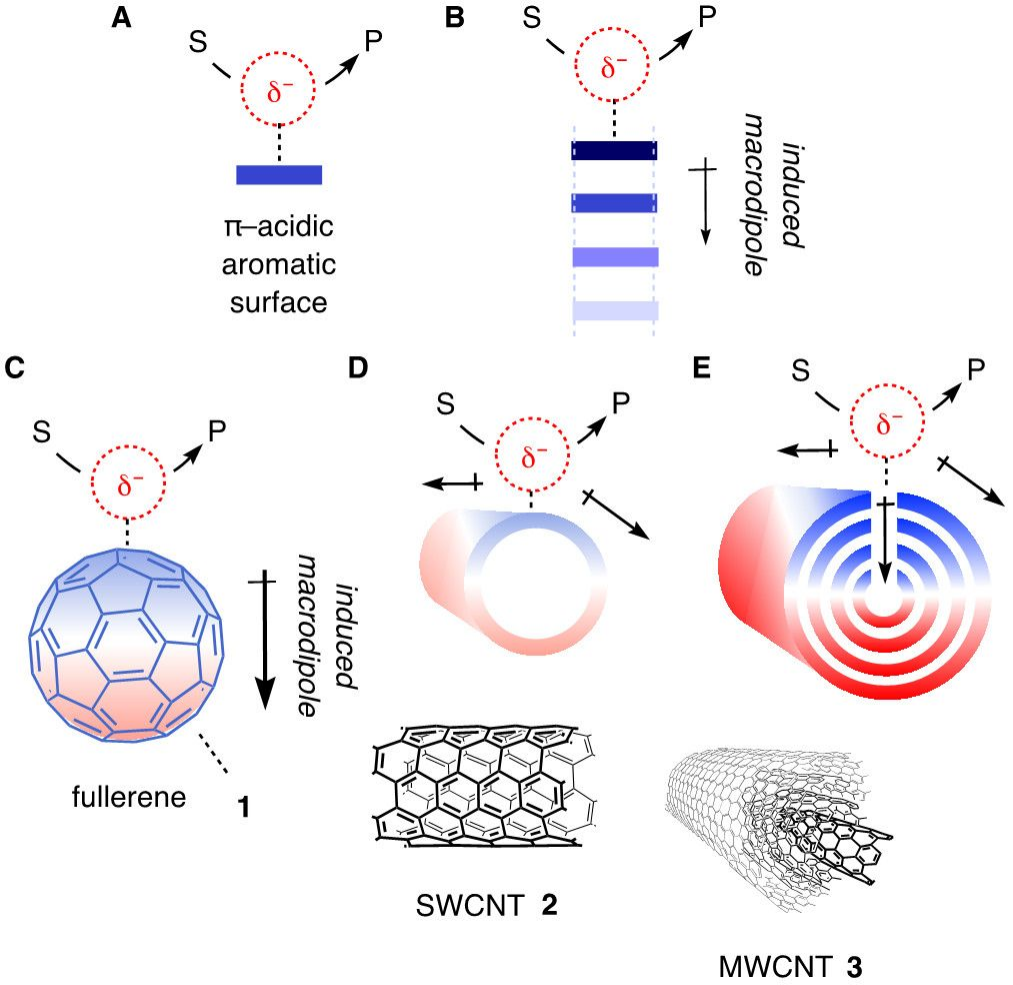
(A) Anion–π catalysis: Stabilization of anionic transition states from substrate S to product P on π-acidic aromatic surfaces. (B) Anion–(π)*_n_*–π catalysis: Stabilization of anionic transition states by polarization of π stacks to induce oriented macrodipoles. (C–E) Anion–π catalysis on (C) C_60_ fullerenes **1**, (D) SWCNTs **2** and (E) MWCNTs **3**. Part E of Figure 1 was adapted from [44] (© 2023 M. A. Gutiérrez López et al., published by the American Association for the Advancement of Science, distributed under the terms of the Creative Commons Attribution 4.0 International License, https://creativecommons.org/licenses/by/4.0).

**Figure 2:**
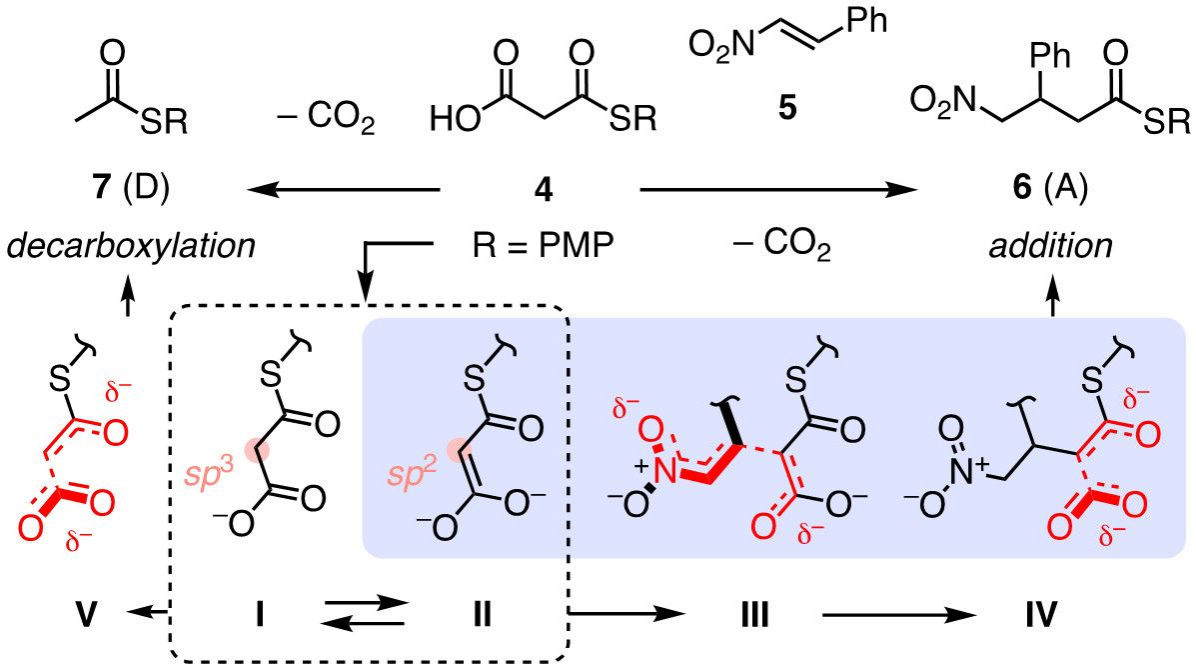
Bioinspired enolate addition chemistry to benchmark anion–π catalysts: Stabilization of “enol” intermediate **II** over “keto” intermediate **I** and nitronate transition state **III** by anion–π interactions (blue) selectively catalyzes the formation of the intrinsically disfavored enolate addition (A) product **6** over decarboxylation (D) product **7**. A/D product ratios are used to characterize anion–π catalysis, increasing A/D ratios indicate increasing anion–π catalysis. PMP = *para*-methoxyphenyl.

Anion–π catalysis was introduced ten years ago [1]. The idea is to stabilize anionic transition states on electron-deficient, π-acidic aromatic surfaces (Figure 1A). The true beginning is arguably in 2015 because it took some time to find the benchmark reaction needed to develop the catalysts (Figure 2) [2]. With this operational enolate chemistry in hand, it quickly became clear that increasing π acidity at the same time decreases the stability of the catalyst [3–5]. This suggested that induced rather than intrinsic anion–π interactions should provide access to really strong catalysts [3]. They have been predicted theoretically to occur on π-stacks [6], and confirmed recently to exist and apply to anion–π catalysis on π-stacked foldamers (Figure 1B) [7] and micelles [8]. However, due to their unique polarizability [9–11], the dream scaffolds for induced anion–π interactions are carbon allotropes. Anionic transition states placed on C_60_ fullerenes **1** will drive the 60 π electrons toward the other side, thus inducing a transient macrodipole that will stabilize the same transition state that induced its formation (Figure 1C) [12]. This intriguing mechanism of catalysis should be further intensified on single-walled carbon nanotubes **2** (SWCNTs, Figure 1D) and multi-walled carbon nanotubes **3** (MWCNTs, Figure 1E) [13]. Multiple substrate/transition-state binding should reduce particularly in-plane polarization of the π system and thus induced anion–π interactions. Since the polarization caused by substrate/transition-state binding hinders additional binding, this effect should occur only at high concentrations.

These expectations were first explored with anion–π catalysis on fullerenes in 2017 [12], followed by SWCNTs and MWCNTs two years later [13]. Particularly MWCNTs have the potential to couple anion–π and cation–π catalysis with electric-field-assisted catalysis [14]. While anion–π (and cation–π [15–16]) catalysis, compared to other unorthodox interactions, has been less impactful than expected [3–4,17–31], this combination has the potential to revolutionize organic catalysis [32–43]. These high expectations have never been realized for technical reasons. However, recent breakthroughs suggest that these methodological problems are now solved [44]. In electrochemical microfluidic reactors, electric-field-assisted anion–π catalysis on MWCNTs **3** is in place to lift anion–π catalysis on a level of general practical significance. For this reason, it appeared timely to recapitulate the results available so far on anion–π catalysis on carbon allotropes.

## Review

### Anion–π catalysis on fullerenes

The use of fullerenes in catalysis is surprisingly underdeveloped [45–51]. Anion–π and cation–π interactions on fullerenes attract similarly little attention until today [52–57]. Anion–π catalysis on fullerenes has been introduced in 2017 [12]. Fullerene anion–π catalysts were developed with the benchmark reaction introduced two years earlier (Figure 2) [2]. In this reaction, at the beginning of all biosynthesis, finetuned malonic acid half thioesters **4** [58–60] are deprotonated, and tautomers **I** and **II** add to an enolate acceptor. For anion–π catalysis, nitroolefins like **5** [60] were convenient acceptors because they are compatible with asymmetric catalysis, and because stabilization of intermediate **III** by privileged nitronate-π interactions drives the reaction forward. Decarboxylation of the resulting intermediate **IV** then affords the chiral addition product **6**. This enolate addition is in kinetic competition with simple decarboxylation, yielding thioacetate **7**. Under most conditions, this decarboxylation is favored. Anion–π catalysis selectively accelerates the intrinsically disfavored but significant enolate addition by stabilizing the planar sp^2^ intermediate **II** that has to add before decarboxylation can occur [2,61]. The non-planar sp^3^ keto intermediate **I** that can decarboxylate through intermediate **V** without preceding enolate addition is less stabilized on the planar π surfaces of anion–π catalysts. The A/D product ratio is thus a convenient measure for anion–π catalysis, the larger the better, with A/D > 1, the intrinsic selectivity for decarboxylation has been inverted [2–3,18].

**Figure 3:**
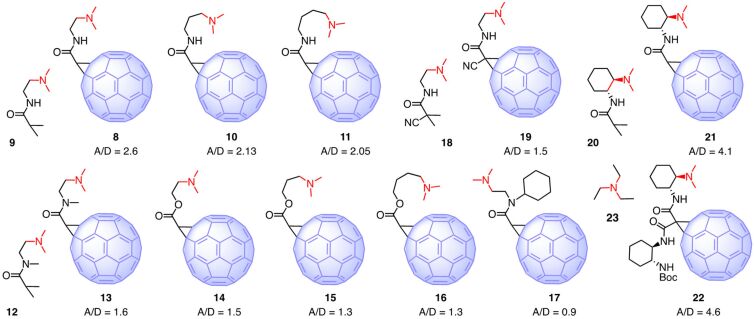
Structure and activity of fullerene-amine dyads to catalyze the intrinsically disfavored but biologically relevant enolate addition chemistry in Figure 2. A/D ratios of fullerene catalysts are normalized against the A/D_0_ ratios of the fullerene-free controls (**9**, **12**, **18**, **20**, **23**), increasing A/D ratios indicate increasing anion–π catalysis on fullerenes.

Applying lessons from simpler anion–π catalysts, trialkylamines were tethered to C_60_ fullerenes [12]. These amines function as bases, their position next to the aromatic surface is essential to turn on anion–π interactions as soon as substrate **4** is deprotonated. Fullerene derivatization with the Bingel reaction installs a cyclopropane that continues with one or two acid derivatives. In fullerene **8**, this is an amide with a short ethylene tether for the tertiary amine (Figure 3).

Fullerene **8** catalyzed the formation of the addition product **6** with high selectivity over the decarboxylation product **7** [12]. The experimental product ratio A/D**_8_** was divided by the product ratio A/D**_9_** measured with the fullerene-free **9**. The resulting A/D**_8_**_/_**_9_** = 2.6 reports the isolate contribution of the fullerene to catalysis in a comparable manner. This high value supported that the high polarizability of fullerenes provides access to strong induced anion–π interactions for efficient anion–π catalysis (Figure 1C).

Elongation of the tether in fullerenes **10** and **11** reduced product ratios to still significant A/D**_10_**_/_**_9_** ~ A/D**_11_**_/_**_9_** = 2.1. The origin of this decrease in anion–π catalysis is likely to include increasing entropy losses in pseudo-macrocyclic transition states. Normalized against the fullerene-free control **12**, a secondary Bingel amide in **13** caused a drop to A/D**_13_**_/_**_12_** = 1.6. Similarly, low A/D**_14_**_/_**_12_** = 1.5 for an ester in **14** supported that removal of the hydrogen-bond donor in **5** rather than steric constraints account for the decrease in anion–π catalysis. Elongation of the tether in the ester series **14**–**16** was as in the amide series from **8** and thus supported entropic contributions to anion–π catalysis. Steric increase of the secondary amide in **17** impeded anion–π catalysis, presumably because the catalytic π surface next to the ammonium cation became inaccessible for anions paired with the tethered ammonium cation. Normalized against the fullerene-free control **18**, an electron-withdrawing cyano group on the cyclopropane in **19** gave similarly poor A/D**_19_**_/_**_18_** = 1.5.

At constant tether length as short as possible, strong increases in anion–π catalysis were found by further minimizing entropy losses in pseudo-macrocyclic transition states, bound non-covalently to both the active π surface and the ammonium cation of the tether. Normalized against the fullerene-free control **20**, the most preorganized dyad **21** gave a new record A/D**_21_**_/_**_20_** = 4.1. This value was much higher than the previous best A/D**_8_**_/_**_9_** = 2.6 with a freely rotating tether. Further increasing activity with fullerene **22** should not be overrated because A/D**_22_**_/_**_23_** = 4.6 was recorded against triethylamine (**23**), which is a less precise control compared to the exactly matching **20**.

### Asymmetric anion–π catalysis on fullerenes

**Figure 4:**
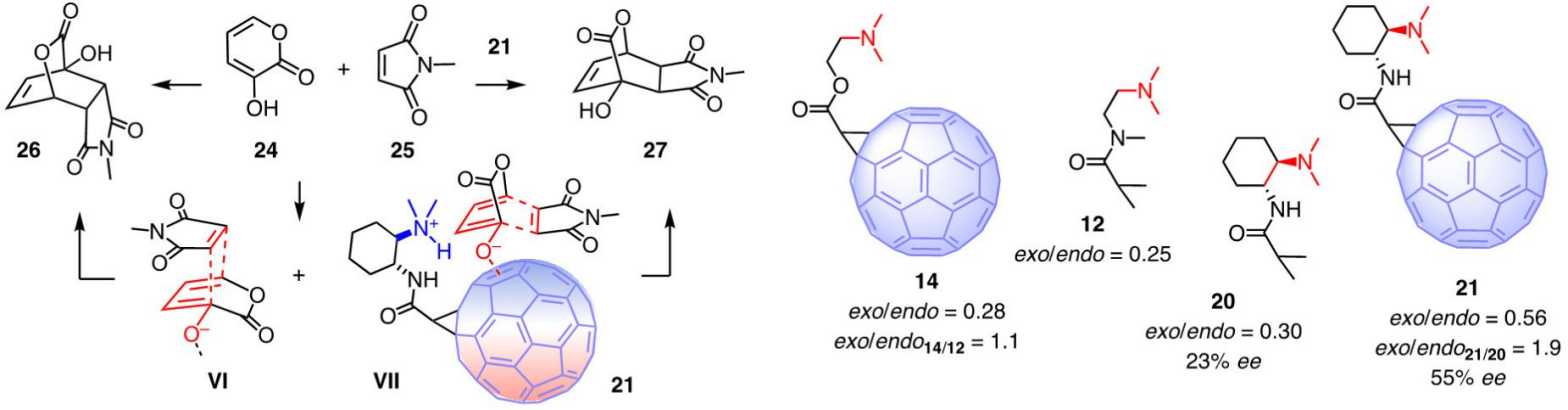
Asymmetric anion–π catalysis of intrinsically disfavored *exo*-selective Diels–Alder reactions on fullerene catalyst **21**, with notional *endo* and *exo* transition states **VI** and **VII**, respectively.

The best fullerene catalyst **21** was applied to other reactions. Diels–Alder reactions are of special interest for anion–π catalysis because of the promise to accelerate an intrinsically disfavored but relevant pathway, like in the benchmark enolate addition (Figure 2) [62]. Namely, in solution, the *endo* transition state **VI** is preferred to maximize orbital overlap (Figure 4) [63]. For π-acidic surfaces, the *exo* transition state **VII** is more completely accessible (Figure 4). The 3-hydroxy-2-pyrone (**24**) was selected as representative diene for the anionic [4 + 2] cycloaddition with maleimide **25** as standard dienophile to afford *endo* product **26** and *exo* product **27** [64–66]. With the less powerful fullerene catalyst **14**, the increase of the *exo*/*endo* selectivity compared to the fullerene-free control **12**, i.e., *exo*/*endo***_14_**_/_**_12_** = 1.1, was negligible [12]. With the best fullerene catalyst **21**, the presence of the fullerene made the diastereoselectivity ratio with *exo*/*endo***_21_**_/_**_20_** = 1.9 nearly doubled. The same was true for the enantioselectivity, which increased from 23% ee for control **20** to 55% ee for anion–π catalyst **21**. These results were in support that stabilization of the intrinsically disfavored *exo* transition state **VII** on the polarizable surface of carbon allotropes can increase diastereo- and enantioselectivity of Diels–Alder reactions.

**Figure 5:**
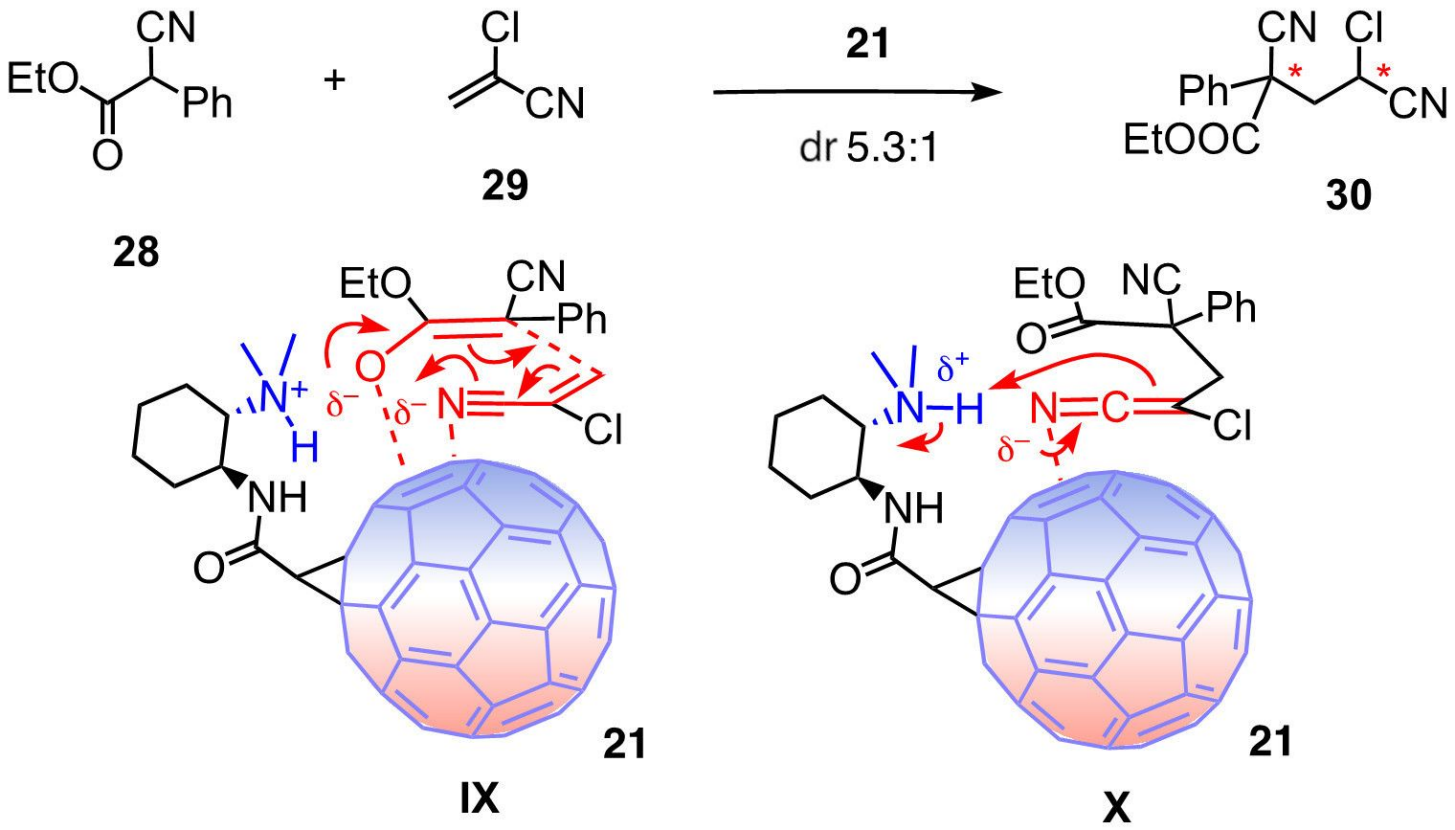
Asymmetric anion–π catalysis to install remote stereogenic centers on fullerene catalyst **21**, with notional transition states **IX** and **X**.

The direct formation of 1,3-nonadjacent stereocenters is a topic of concern in asymmetric catalysis [67]. To explore compatibility with anion–π catalysis, the addition of ethyl 2-cyano-2-phenylacetate (**28**) to 2-chloroacrylonitrile (**29**) was selected (Figure 5) [68]. In the presence of 5 mol % of the best fullerene catalyst **21**, conversion into dicyanide **30** reached 72% within 5 days at ambient temperature [67]. While enantioselectivity was negligible, dr 5.3:1 was the best diastereoselectivity among all tested anion–π catalysts. This result was consistent with the stabilization of the anionic intermediates **IX** and **X** and the respective transition states on the polarized fullerene surface.

### Anion–π autocatalysis on fullerenes

**Figure 6:**
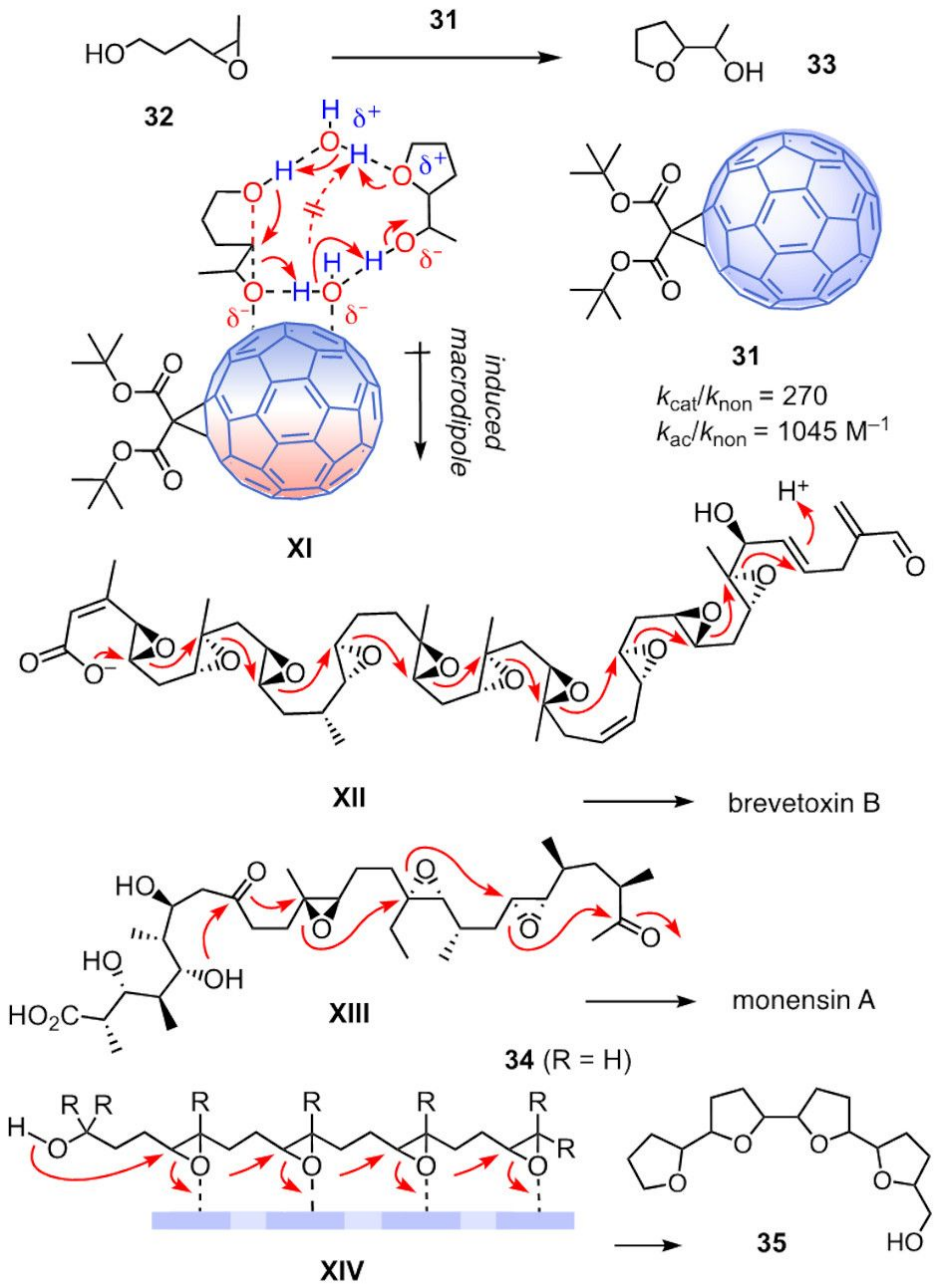
Primary anion–π autocatalysis on monofunctional fullerene **31**, with catalytic and autocatalytic rate enhancements, representative cascade cyclizations with anti-Baldwin (**XII**) and Baldwin (**XIII**) selectivity in nature, and primary anion–π autocatalysis of cascade cyclization **XIV** on catalysts other than carbon allotropes.

The autocatalysis of epoxide-opening ether cyclization on π-acidic aromatic surfaces has been identified in 2018 as an emergent property of anion–π catalysis [69]. In this series, fullerene catalyst **31** was found to catalyze the cyclization of epoxide **32** into THF **33** (Figure 6). The rate enhancement for catalysis was 270, whereas autocatalysis accelerated the reaction by 1045 M^−1^. The origin of autocatalysis on π-acidic surfaces was clarified only this year because the involvement of two molecules of water in the decisive transition state **XI** complicated the situation [70].

This reaction was intriguing for reasons beyond autocatalysis. It is the only example so far where fullerenes act as anion–π catalyst without additional activating groups, usually a tethered base to inject a negative charge into the substrate directly on top of the catalytic π surface [69]. The *tert*-butyl esters in Bingel fullerene **31** serve only to improve solubility and are not expected to participate in catalysis. Moreover, epoxide opening polyether cyclizations are among the most impressive cascade reactions in nature [71–73]. Best known is the hypothetical cascade **XII** in the biosynthesis of brevetoxin B [74]. It affords eleven fused ethers by violating the Eschenmoser–Dunitz–Baldwin guidelines [75–78] in every step. Among the Baldwin compatible examples from nature, the cascade **XIII** leading to monensin A is arguably the most popular [79]. On anion–π catalysts other than carbon allotropes, up to four epoxides **34** have been cyclized into the monensin-like tetra-THF **35** as outlined in intermediate **XIV** [24–25]. Thus, this reaction can be considered as the anion–π catalysis counterpart of the steroid cyclizations catalyzed in nature with the charge inverted, conventional cation–π interactions [15].

### Anion–π catalysis on fullerene dimers

**Figure 7:**
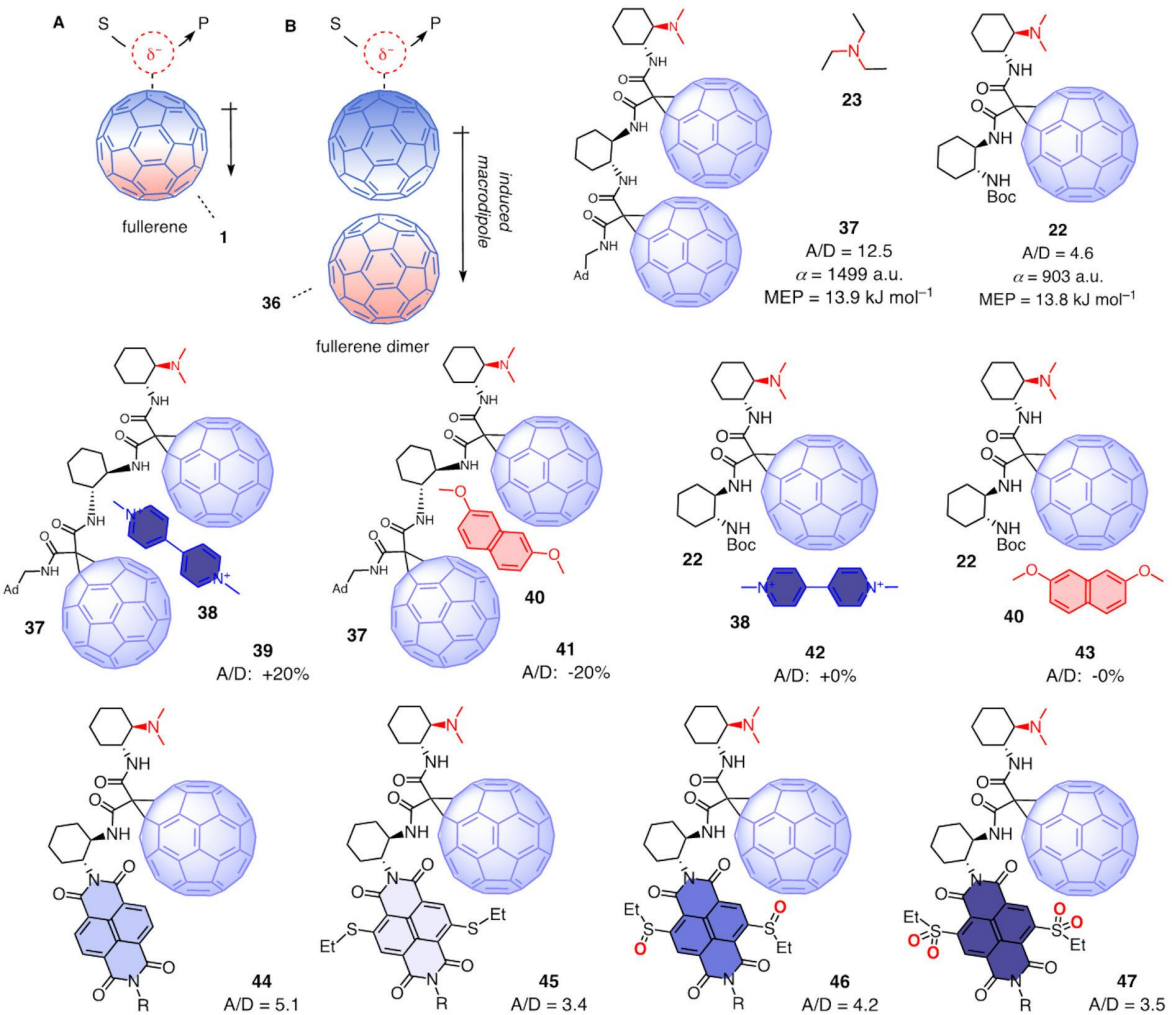
(A) Macrodipoles induced by anionic transition states account for anion–π catalysis on fullerenes. (B) Fullerene dimers double polarizability of the π system and thus anion–π catalysis, with structure of fullerene dimer **37**, intercalating activators and inactivators, monomeric fullerene and fullerene-NDI controls, and A/D ratios for products **6** and **7** normalized against the A/D_0_ ratio of the fullerene-free control **23**, increasing A/D ratios indicate increasing anion-π catalysis.

The strength of anion–π interactions increases with face-to-face π stacking because the delocalized π electrons move within the stack away from the charge, which induces a macrodipole along the stack that supports the binding of the anion (Figure 1B) [61]. What works for anion–(π)*_n_*–π catalysis on π-stacked foldamers [61] and micelles [8] should apply to fullerenes as well. The unique polarizability of fullerene monomers like **1** is thought to induce strong anion–π interactions and thus account for the observed catalytic activity (Figure 7A) [12]. This polarizability should further increase in fullerene dimers like **36** [80]. Anions, anionic reactive intermediates and anionic transition states on top of fullerenes dimers should thus induce a larger macrodipole which should in turn strengthen their binding to the expanded π system and thus increase anion–π catalysis (Figure 1B).

Fullerene dimer **37** was equipped with the tethered tertiary amine needed to deprotonate the substrate **4** and produce the conjugate bases **I** and **II** as the first reactive intermediates directly on the active π surface (Figure 2 and Figure 7) [80]. Calibrated against triethylamine **23**, fullerene dimer **37** catalyzed enolate addition with an A/D**_37_**_/_**_23_** = 12.5. This is more than twice the A/D**_22_**_/_**_23_** = 4.6 of the respective monomer **22**, which was already best among fullerene monomers. In computational simulations, the positive maximum of the MEP surface next to the amine base was the same for dimer **37** (+13.9 kJ mol^−1^) and monomer **22** (+13.8 kJ mol^−1^), supporting that the dramatic increase in activity did not originate from a change in intrinsic anion–π interactions. The computed polarizability of dimer **37** (α = 1499 a.u.) was almost twice as high as that of monomer **22** (α = 903 a.u.), confirming that powerful induced anion–π interactions account for the high catalytic activity.

Twenty equivalents of the π-acidic intercalator **38** increased the catalytic activity of fullerene dimer **37** by 20%. This increase was consistent with the formation of the π-stacked complex **39** with increased polarizability and electron deficiency. With the π-basic intercalator **40**, the catalytic activity of fullerene dimer **37** decreased by 20%. This complementary decrease supported the formation of complex **41** with decreased electron deficiency. Control experiments with fullerene monomer **22** gave unchanged catalytic activity with intercalators **38** or **40**, which suggested that complexes **42** and **43** do not form. The insensitivity of fullerene monomers **22** thus supported that **38** and **40** activate and inactivate fullerene dimers **37** by intercalation in-between the two fullerenes.

Replacement of the second fullerene in dimer **37** with a poorly polarizable naphthalenediimide (NDI) in **44** increased the catalytic activity of fullerene monomer **22** much less significantly. With less electron-deficient NDIs carrying two sulfide donors in the core, the catalytic activity of **45** dropped below that of fullerene monomer **22**. Oxidation of the sulfide donors into sulfoxide acceptors increased the catalytic activity much less than expected, resting below that of unsubstituted NDIs despite stronger π acidity. Supported by experimental and computational data, this poor performance of dyad **46** was explained by lone-pair π interactions of the fullerene with the donating oxygens. These lone-pair π interactions were even more impactful on the sulfone level. Compared to the sulfoxides in **46**, the catalytic activity of dyad **47** decreased rather than increased despite stronger π acidity.

Supported by computational predictions [80–81], record activities in anion–π catalysis with fullerene dimers called for higher oligomers. However, synthetic efforts were not fruitful, mostly due to poor solubility.

### Anion–π catalysis on carbon nanotubes

With fullerenes confirmed as privileged scaffold for induced anion–π catalysis but higher oligomers inaccessible [12,67,80], the obvious next move was to switch to carbon nanotubes. Compared to the sixty free electrons rushing toward one side of a C_60_ fullerene **1** to create a large macrodipole in response to an anionic transition state, the number of electrons available to maximize polarizability already in SWCNTs **2** is much higher (Figure 1D) [9–11]. Continuing in this series to increase polarizability with carbon allotropes, MWCNTs **3** emerge as privileged scaffold for anion–π catalysis because polarizability is possible not only along the tubes but also between the nanotubes, like in π-stacked foldamers (Figure 1E).

**Figure 8:**
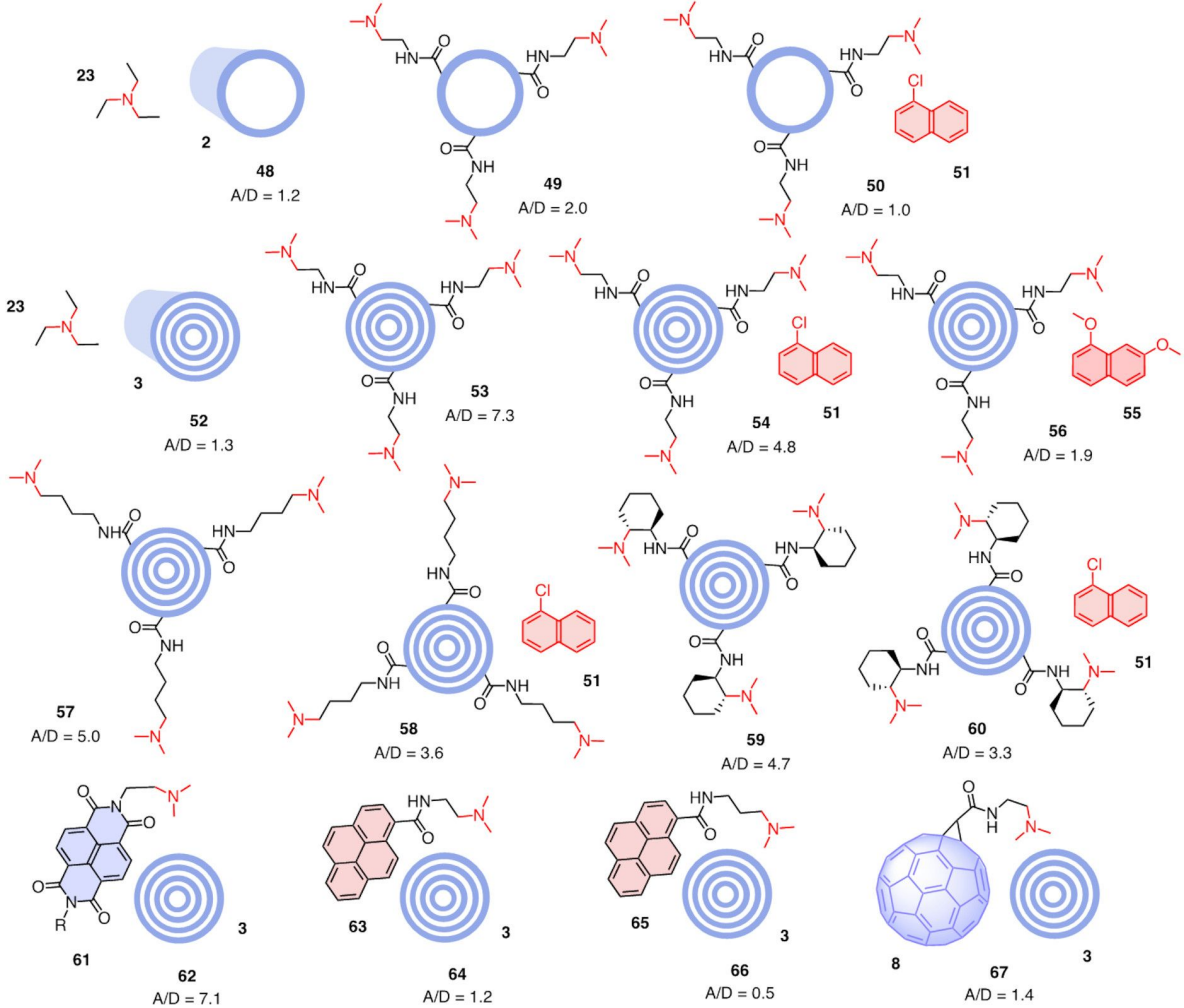
Structure and activity of covalently and non-covalently modified SWCNTs and MWCNTs, with A/D ratios for products **6** and **7** normalized against the A/D_0_ ratios of the CNT-free controls, increasing A/D ratios indicate increasing anion–π catalysis.

Numerous reports on catalysis with carbon nanotubes have appeared [45–46,82–94]. They serve as catch-and-release scaffolds in different variations, and, less frequently, as (photo)redox partners. Although they might contribute to these activities, anion–π interactions have not been considered. Anion–π catalysis on carbon nanotubes has been introduced explicitly in 2019 [13]. Already in the presence of pristine SWCNTs **2**, the ability of TEA **23** to catalyze enolate addition with **4** increased to A/D**_48_**_/_**_23_** = 1.2 for a virtual catalytic complex **48** between the two (Figure 8). Covalent modification of SWCNTs with tertiary amines as in **49** further increased activity to A/D**_49_**_/_**_23_** = 2.0. Suppression of this increase in a virtual complex **50** with a competitive inhibitor **51** further supported that the observed activity originates from anion–π catalysis.

With A/D**_52_**_/_**_23_** = 1.3, pristine MWCNTs **3** failed to increase the activity of TEA **23** much more than SWCNTs **2**. This is not surprising because virtual complexes **52** and **48** are not expected to exist to an appreciable extent. With covalent modification, MWCNTs **53** with A/D**_53_**_/_**_23_** = 7.3 outperformed the corresponding SWCNTs **49** with A/D**_49_**_/_**_23_** = 2.0 clearly. This significant increase in activity was consistent with the increase in polarizability from SWCNTs **2** to MWCNTs **3** (Figure 1). Compared to fullerene monomers and dimers, this activity cannot be overestimated because MWCNTs operate in suspension rather than solution, that is as formal heterogenous rather than homogenous catalysts.

Inhibition of the covalently modified MWCNT **53** with inhibitor **51** in complex **54** was supported by a drop from A/D**_53_**_/_**_23_** = 7.3 to A/D**_54_**_/_**_23_** = 4.8. Under identical conditions, the much more π-basic inhibitor **55** inhibited enolate addition by the MWCNT **53** almost completely, i.e., A/D**_56_**_/_**_23_** = 1.9 for the formal catalyst-inhibitor complex **56**. Increasing inhibition with the π basicity of the inhibitor was consistent with powerful anion–π interactions accessible on MWCNTs for efficient anion–π catalysis.

Like in the fullerene series, elongation of the tether in **57** decreased catalytic activity to still important A/D**_57_**_/_**_23_** = 5.0, presumably due to entropic reasons. The activity of inhibitor **51** remained detectable under standard conditions, thus supporting the formation of complex **58** with A/D**_58_**_/_**_23_** = 3.6. Unlike the fullerene series, preorganization of the tight tether in MWCNT **59** with A/D**_59_**_/_**_23_** = 4.7 did not give the best activity. This suggested that with the fully rigid and tight tether, small differences in local environment turned an advantageous match with the most convex surface of fullerene **21** into a slight mismatch with MWCNTs **59**. However, activities remained high and inhibition with inhibitor **51** in formal complex **60** was with A/D**_60_**_/_**_23_** = 3.3 preserved, also with this mildly mismatched tether.

The π-acidic NDI **61** with a Leonard-turned tertiary amine is a privileged small-molecule anion–π catalyst that catalyzes the enolate addition of malonate **4** with an activity that, however, does not reach that of the best carbon allotropes [95]. In the presence of pristine MWCNTs **3**, the activity of the NDI catalyst **61** increased significantly, implying the formation of complex **62** with A/D**_62_**_/_**_61_** = 7.1. While the reaction presumably still occurred on the NDI surface, this very high A/D value suggested that efficient face-to-face stacking connected the active NDI surfaces to the nanotube to increase intrinsic anion–π interactions with the highest polarizability.

For interfacing with CNTs, the π-basic pyrenes rather than the π-acidic NDIs are most popular [82,88,90,96–99]. Equipped with a tertiary amine, the catalytic activity of dyad **63** increased only slightly in the presence of MWCNTs **3**, suggesting that the formed π-basic complex **64** is with A/D**_64_**_/_**_63_** = 1.2 much less active than the π-acidic NDI complex **62** with A/D**_62_**_/_**_61_** = 7.1, which is as expected for operational anion–π catalysis. With a longer tether, the already weak activity of pyrene **65** even decreased rather than increased in the presence of MWCNTs **3**, with a formal complex **66** operating with A/D**_66_**_/_**_65_** = 0.5. The activity of the fullerene catalyst **8** did not increase much in the presence of MWCNTs **3**, giving formal complex **67** with A/D**_67_**_/_**_8_** = 1.4. This was meaningful because the high activity of fullerene **8** with A/D**_8_**_/_**_9_** = 2.6 (Figure 3) leaves little room to improve, and the interaction between convex aromatic surfaces is not favorable.

Taken together, anion–π catalysis on multiwalled carbon nanotubes (**53**, A/D = 7.3) is better than on single-walled carbon nanotubes (**49**, A/D = 2.0) and monomeric fullerenes (**22**, A/D = 4.6) but weaker than on fullerene dimers (**37**, A/D = 12.5). The true activity is presumably much higher because MWCNTs, and SWCNTs, are heterogenous catalysts that operate as suspensions with only a minor fraction of the total π surface accessible for function. Fullerene monomers and dimers, in contrast, are homogenous catalysts that are fully dissolved and thus fully accessible. Anion–π catalysis thus increases roughly with the polarizability of the carbon allotrope. The overall less convincing activities found with SWCNTs could originate from more favorable weakening of the exclusively in-plane polarization by multiple substrate/transition-state binding under the selected experimental conditions. Besides these overall minor reservations for SWCMTs, the found activities are among the best observed [3], confirming the promise of induced anion–π interactions on carbon allotropes for catalysis.

**Figure 9:**
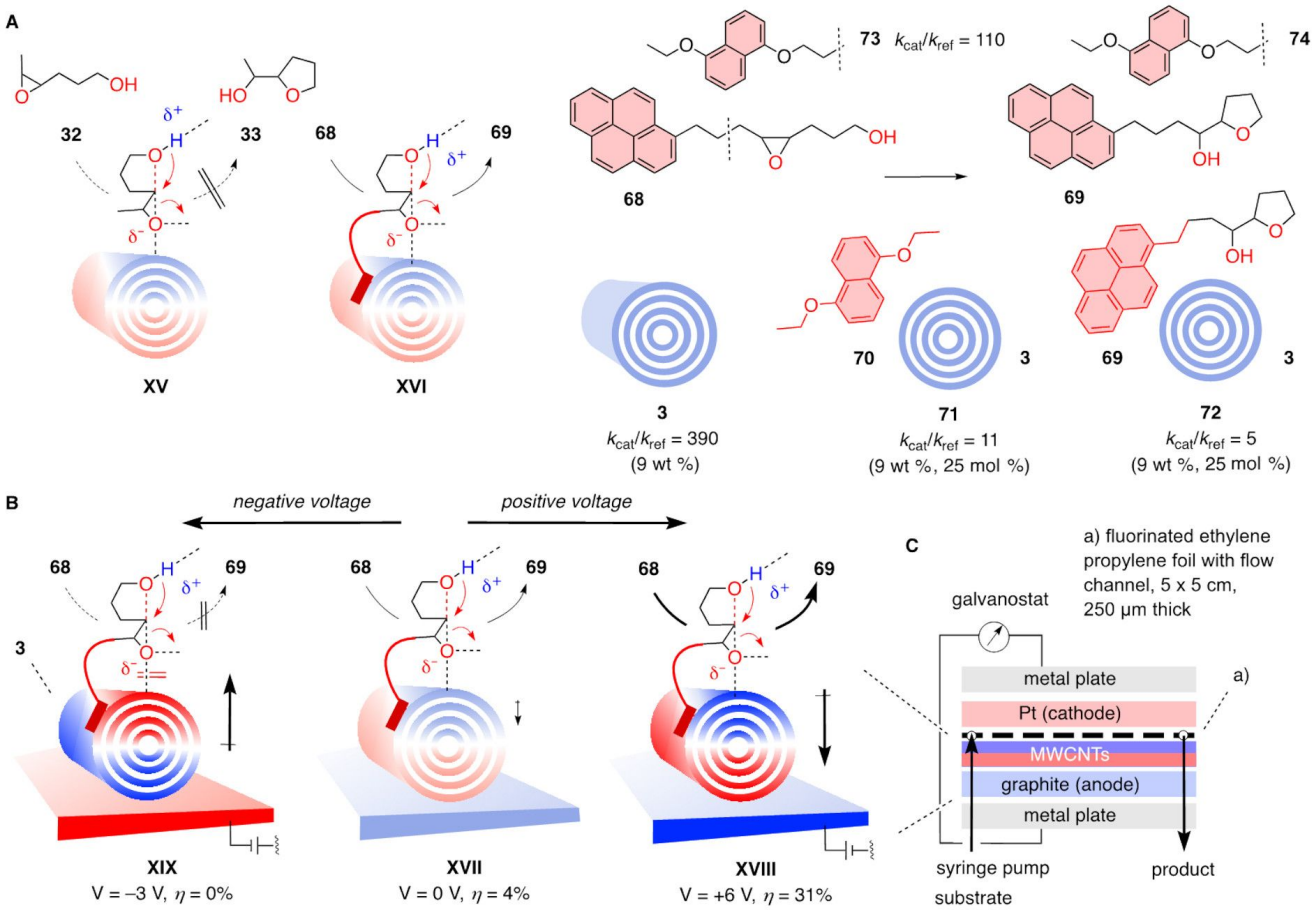
(A) Epoxide-opening ether cyclization on pristine carbon nanotubes occurs with (**XVI**) but not without (**XV**) interfacers such as pyrene in **68** or DAN in **73**, catalyst inactivation with **70** in formal complex **71** and product **69** in formal complex **72** supports anion-π catalysis but not autocatalysis. (B) Electric-field-induced anion–π catalysis on MWCNTs **3** in electrochemical microfluidic reactors, with reactor design (C) and notional transition states for ether cyclization of **68** at positive (**XVIII**), zero (**XVII**) and negative voltage (**XIX**). Parts B and C of Figure 9 were adapted from [44] (© 2023 M. A. Gutiérrez López et al., published by the American Association for the Advancement of Science, distributed under the terms of the Creative Commons Attribution 4.0 International License, https://creativecommons.org/licenses/by/4.0).

In contrast to these conclusions made with modified carbon nanotubes, epoxide-opening ether cyclization of **32** occurred on undermodified fullerene **31** (Figure 6) but not on pristine MWCNTs **3** (Figure 9). Modified carbon nanotubes exert their catalytic power because the tethered base injects the negative charge into the substrate right on top of the polarizable π surface of the tube. Inability of pristine MWCNTs **3** to open and cyclize epoxide **32** thus implied insufficient substrate binding to form substrate-catalyst complexes that can proceed to transition state **XV** (Figure 9A). To increase binding, substrates would have to be equipped with interfacers. For MWCNTs **3**, interfacing with pyrene is most popular [82,88,90,96–99]. A pyrene interfacer was thus attached to substrate **32** [44]. Binding of the pyrene in the resulting substrate **68** to the MWCNT catalyst **3** should then afford a substrate-catalyst complex strong enough to access the interfaced transition state **XVI** and yield product **69**. Already the presence of 9 wt % of MWCNT suspensions gave rate enhancements of 390. They increased with increasing MWCNT concentrations.

In the presence of 25 mol % of inhibitor **70**, rate enhancements dropped to 11. This decrease was consistent with the formation of the formal inhibitor-catalyst complex **71**, where the π-basic DANs **70** stacked onto MWCNTs hinder the access of substrate **68**, physically, electrostatically, or both. The addition of 25 mol % product **69** at the beginning of the reaction decelerated rather than accelerated the cyclization from rate enhancements of 390 down to 5. The formal product-catalyst complexes **72** were, however, weak enough not to interfere with full substrate conversion, that is turnover. They provided support for the occurrence of anion–π catalysis but not autocatalysis on MWCNTs. The disappearance of autocatalysis on MWCNTs provided consistent support that the polarization of the extended π system induced by substrate/transition-state binding hinders additional binding. An anionic transition state on the extended π surface thus not only induces its own stabilization by polarization, i.e., self-creates its own catalyst, but also self-protects against its destruction by multiple binding at sufficiently low concentrations (Figure 1).

Rate enhancements for the conversion of substrate **73** into product **74** in the presence of 9 wt % pristine MWCNTs **3** were not as high as with substrate **68**. This result was consistent with pyrene as best interfacers for MWCNTs. For anion–π catalysis on NDI stacks, the reversed order was obtained, consistent with the preferred formation of DAN-NDI charge transfer complexes [8,100–106].

The efficient conversion of interfaced substrates **68** and **73** on pristine MWCNTs **3** was of particular interest with regard to catalysis initiated by oriented external electric fields (OEEFs, Figure 9B). The idea of OEEF catalysis has been around for a long time as a promising, bioinspired concept to revolutionize organic synthesis on the broadest sense [32–35]. Indeed, every chemical transformation can be seen as a directional displacement of point charges, electrons. Control over speed and direction of this charge translocation by OEEFs should allow to generally manipulate molecular transformation, from speed to selectivity and access to completely new reactions. Internal electric fields have been shown to account for much of the power of enzymes [41–43]. The translation of these lessons from nature into OEEF catalysis has so far been slow for a series of most demanding challenges [32–40]. It has been shown that anion–π catalysis with NDIs on ITO electrodes could solve some but not all of these challenges, and relevance for practice remained negligible [14].

Operational anion–π catalysis on MWCNTs fundamentally changed this situation. Integrated into electrochemical microfluidic reactors [107–110], this breakthrough promised to solve all problems obstructing the use of OEEF catalysis in a remarkably straightforward manner. Electrochemical microfluidic reactors should provide access to strong fields at voltages low enough to avoid electron transfer, offer high enough effective catalyst to substrate ratios and work without interfering electrolytes (Figure 9C). MWCNTs drop casted on graphite anodes [111] then should translate the applied OEEFs into strong local macrodipoles. These oriented macrodipoles, depending on the sign of the applied voltages, should then enable strong anion–π and cation–π interactions [112–115] and accelerate and direct the electron displacement during the reaction. Possible limitations at high concentrations in suspension from catalyst depolarization by multiple binding (Figure 1) naturally do not apply to permanent polarization by OEEFs (Figure 9B).

To elaborate on these great expectations, pristine MWCNTs **3** were drop casted on the graphite anodes of electrochemical microfluidic reactors [44]. The substrate was injected by a syringe pump, the product was collected at the other end of the microflow channel and analyzed by HPLC (Figure 9C). Without applied voltage, the reaction essentially did not occur under these heterogenous conditions. This inactivity indicated that transition state **XVII**, characterized by interfacing and induced MWCNT polarization by the transition state itself but not from OEEFs, is hardly accessible (Figure 9B). With increasing positive voltage applied, conversion increased in a pseudo-linear manner. At 6 V, the conversion after one passage through the system at a flow rate *Q*_v_ = 25 µL min^–1^ increased to 31%. The emergence of conversion in response to applied voltage was consistent with operational OEEF catalysis, that is the stabilization of transition state **XVIII** by the local oriented macrodipole of MWCNTs that are polarized by the OEEF applied. Inversion of the applied voltage to –3 V removed the minimal activity present without voltage. This result was important because it was not only consistent with the existence of OEEF catalysis with a destabilized transition state **XIX**. It also disfavored contributions from S_N_1-type mechanisms and, most important, from electron transfer.

### Anion–π catalysis on graphite

**Figure 10:**
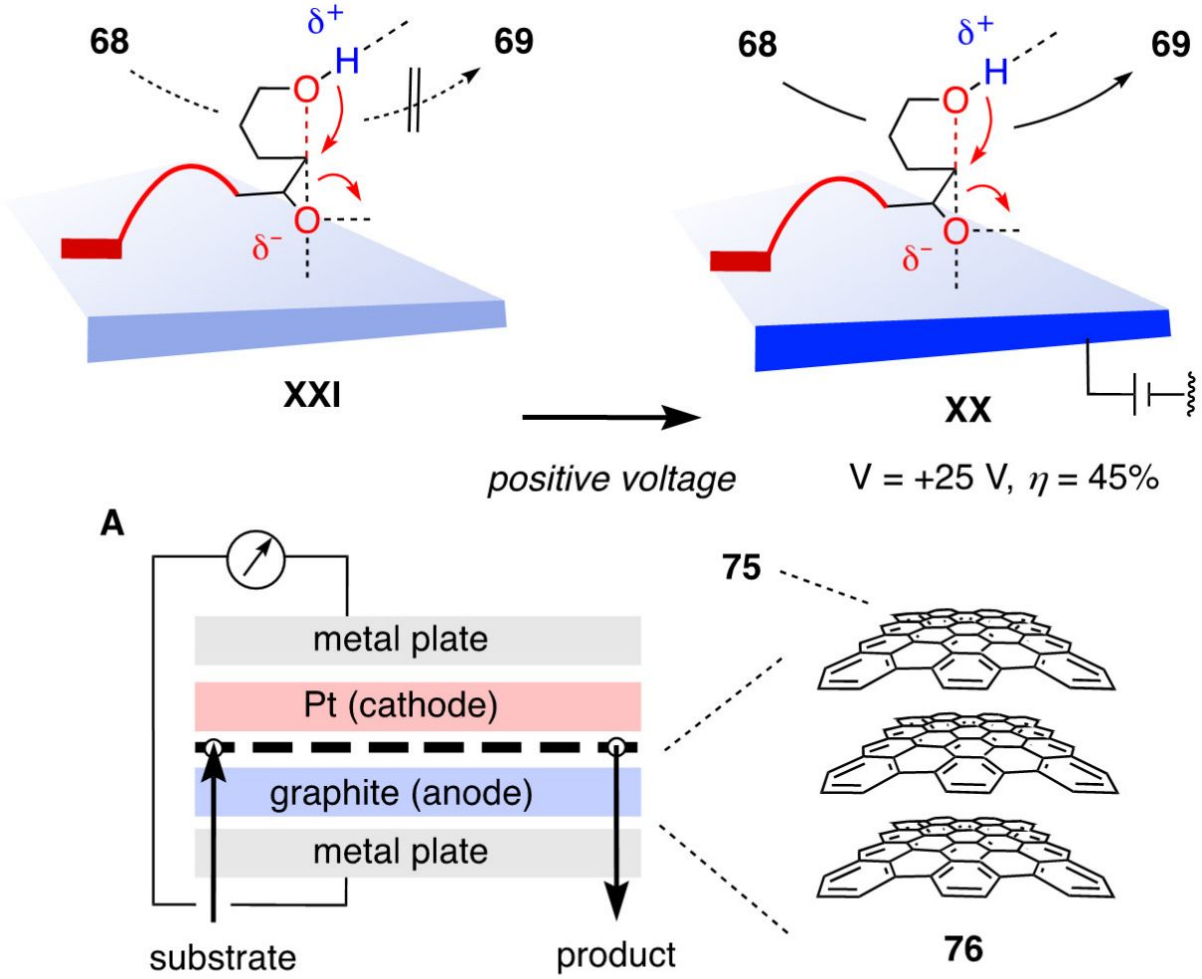
Electric-field-induced anion–π catalysis on MWCNTs **3** on graphite **76** in electrochemical microfluidic reactors, with reactor design (A) and notional transition states for ether cyclization of **68** at positive (**XX**) and zero voltage (**XXI**) on graphite **76**. Figure 10 was adapted from [44] (© 2023 M. A. Gutiérrez López et al., published by the American Association for the Advancement of Science, distributed under the terms of the Creative Commons Attribution 4.0 International License, https://creativecommons.org/licenses/by/4.0).

Beginning with spherical fullerenes, expansion of the aromatic surface of carbon allotropes leads to SWCNTs and MWCNTs. Further expansion by unrolling nanotubes into infinite sheets leads to graphene **75** as formal homolog of SWCNTs **2** and graphite **76** as formal homolog of MWCNTs **3** (Figure 10) [112,114–123]. Graphite is the oldest, most common carbon allotrope composed of sp^2^ hybridized atoms, complementary to diamond as the archetypal sp^3^ carbon allotrope. Conductive because of the delocalized electrons of the giant π system, graphite is commonly used as electrode. With this giant π system, graphite qualifies as potential anion–π and cation–π catalyst, depending on the orientation of the planes in the solid.

Also in electromicrofluidic reactors, graphite electrodes are used. It was thus tempting to try OEEF-induced anion–π catalysis in the absence of MWCNTs, directly on graphite. However, without MWCNTs drop casted on the graphite electrode, the conductivity of the reactor was much lower [44]. This meant that already small currents produce high voltage. Despite this undesirable situation, conversion of **68** was observed to increase with increasing voltage. This observation was particularly intriguing because electric-field-induced anion–π catalysis on graphite might be relevant with regard to early steps in the origin of life [124–125].

## Conclusion

Anion–π catalysis on carbon allotropes originates from the observation that access to strong anion–π interactions by increasing the intrinsic π acidity of aromatic surfaces is not realistic. Any permanent withdrawal of electron density destabilizes the aromatic system before anion–π interactions would become really attractive for catalysis [3]. This observation called for a shift of attention from intrinsic to induced anion–π interactions. Polarizability of the aromatic system has been predicted since the beginning to provide access to really strong anion–π interactions [61]. For anion–π catalysis, the shift from intrinsic to induced anion–π interactions suggests that the anionic transition state will induce the formation of its own catalyst. Close to a polarizable π surface, such an anionic transition state will drive all movable electrons away. This induced charge relocation will generate strong macrodipoles which are oriented to stabilize the same anionic transition state that induces their formation (Figure 1).

The shift of attention from intrinsic to induced anion–π interactions thus called for aromatic systems of highest polarizability, that is carbon allotropes [9–11]. This account recapitulates how anion–π catalysis on carbon allotropes was explored first on fullerene monomers, then fullerene dimers, SWCNTs and MWCNTs. Studies mainly focusing on enolate addition chemistry showed that selectivity generally increases with the polarizability of the carbon allotrope. Other reactions like asymmetric anion Diels-Alder reactions, the construction of 1,3-nonadjacent stereocenters and bioinspired ether cyclizations have been realized as well.

The emerging combination with oriented external electric fields changes the mechanism of anion–π catalysis on carbon allotropes [44]. Rather than an anionic transition state creating its own catalyst, the OEEF polarizes the carbon allotrope in advance. The resulting macrodipoles then should enable strong anion–π and cation–π interactions depending on their orientation, and accelerate and direct electron displacement during the reaction. This translation of the external field into local oriented macrodipoles solves one important challenge that has delayed progress with OEEF-induced catalysis. Namely, the fields predicted to accelerate and direct the flow of electrons during a reaction are much larger than the voltage needed to turn-on electron transfer and redox chemistry. This dilemma is overcome by carbon allotropes. They translate voltages weak enough to avoid electron transfer into oriented local molecular macrodipoles that are strong enough to access significant anion–π and cation–π interactions and thus accelerate electrons movement during a reaction.

The rich collection of additional problems holding back progress with OEEF-induced catalysis could be addressed with electrochemical microfluidic reactors. Most important are high effective catalyst to substrate ratios, high fields from small voltages, and no need to add electrolytes. These electrochemical microfluidic reactors have been constructed for a completely different purpose, that is practical access to organic redox chemistry [107–110]. Our results suggest that there might be more to win before the electrons jump. Being general and easy to use, the introduced supramolecular organocatalytic systems promise to lift all involved topics to a new level of significance. Essentially every reaction consists of the movement of electrons, from nucleophile to electrophile. To accelerate and direct this charge displacement, any electron-rich motif in transition states and reactive intermediates should be stabilizable by induced anion–π interactions with MWCNTs that are polarized by an electric field. Inversion of the applied voltage should allow to stabilize the respective electron-poor motifs by induced cation–π interactions, and the combination with co-catalysts interfaced on MWCNTs should provide general access to asymmetric catalysis. OEEF-induced anion–π and cation–π catalysis on carbon allotropes, affordable, clean and general, thus have the potential to non-covalently electrify organic synthesis in the broadest sense.
